# Editorial: Protein-based nanocarriers for delivery of nutraceutical, diagnostic and therapeutic agents

**DOI:** 10.3389/fphar.2022.991474

**Published:** 2022-08-08

**Authors:** Jian Zhong, Ruirui Qiao, Wei-En Yuan

**Affiliations:** ^1^ Xinhua Hospital, Shanghai Institute for Pediatric Research, Shanghai Key Laboratory of Pediatric Gastroenterology and Nutrition, Shanghai Jiao Tong University School of Medicine, Shanghai, China; ^2^ National R&D Branch Center for Freshwater Aquatic Products Processing Technology (Shanghai), Integrated Scientific Research Base on Comprehensive Utilization Technology for By-Products of Aquatic Product Processing, Ministry of Agriculture and Rural Affairs of the People’s Republic of China, Shanghai Engineering Research Center of Aquatic-Product Processing and Preservation, College of Food Science and Technology, Shanghai Ocean University, Shanghai, China; ^3^ Australian Institute for Bioengineering and Nanotechnology, The University of Queensland, Brisbane, QLD, Australia; ^4^ Engineering Research Center of Cell and Therapeutic Antibody, Ministry of Education, School of Pharmacy, Shanghai Jiao Tong University, Shanghai, China

**Keywords:** delivery, lipid, nanocarriers, protein, polymer

In the past several decades, with the advent of nanotechnology, nanocarriers have become one of the most important and promising delivery systems for nutraceutical, diagnostic and therapeutic agents. Nanocarriers are generally defined as nanoscale particle systems with well-defined physicochemical properties for encapsulating nutraceutical, diagnostic and therapeutic agents (e.g., chemotherapeutics, inhibitors, nucleic acids, proteins, etc.) with the purpose of prolonging their blood circulation times and delivering them into the target site with a controlled release. Nanocarriers have become well appreciated in recent years due to their unique structural, chemical, mechanical, magnetic, electrical, and biological properties. Typically applied nutraceutical, diagnostic and therapeutic agents include nutraceutical agents, contrast agents, chemotherapy drugs, inhibitors, nucleic acids, proteins, etc. According to the chemical nature, the most widely studied nanocarriers can be classified into organic, inorganic, polymeric (e.g., liposomes, dendrimers, and micelles), and biological nanostructures (e.g., proteins). It is considered a great advantage of nanocarriers over other carriers since it allows the use of nutraceutical, diagnostic and therapeutic agents that are not stable in their free form to form stable nanocarriers. In recent years, there is a growing interest in developing protein-based nanocarriers as they are non-toxic and biodegradable, which is essential in drug delivery applications. Additionally, proteins have exceptional characteristics such as bioavailability, biodegradability, nonantigenicity, high nutritional value, sufficient sources, and extraordinary binding capacity of various nutraceutical, diagnostic and therapeutic agents. The shape of protein nanoparticles can be classified into undeformable solid spherical nanoparticles, nanofibrils, nanotubes, nanogels, nanocages, and plate-shaped nanoparticles. Protein nanoparticles can be used to construct different interactions between proteins and encapsulated agents to form three-dimensional networks, which can offer a variety of possibilities such as reversible protection and specific target delivery. Protein-based nanocarriers can be prepared by animal proteins (e.g., gelatin, collagen, albumin, milk proteins, silk proteins, and elastin) and plant proteins (e.g., zein, gliadin, soy proteins, and lectins). Protein-based nanocarriers can be prepared by a variety of methods such as desolvation, coacervation, emulsification, nanoprecipitation, nanospray drying, self-assembly, electrospraying, and crosslinking. Therefore, the research and development of protein-based nanocarriers is a hotspot in the field of functional foods, diagnostics, and therapeutics. Nanopharmacology is a Frontier and rapidly growing branch of pharmacology to study the interaction of a nanomedicine with living systems at the nanoscale level. The *in vitro* and *in vivo* nanopharmacological properties of protein-based nanocarriers can be affected by their physicochemical properties such as protein composition, protein solubility, properties of nutraceutical, diagnostic and therapeutic agents, and nanocarrier surface properties. Therefore, it is important to explore the relationship between physicochemical properties and nanopharmacological properties of protein-based nanocarriers.

This Research Topic is a platform to discuss protein-based nanocarriers for the delivery of nutraceutical, diagnostic and therapeutic agents. Chaubey et al. reviewed ligand-anchored polymers for drug targeting in the treatment of colonic disorders. This review discussed dosage form developments, colonic drug delivery, traditional drug delivery methods in colon disorders, the need for ligand-based colon-targeted drug delivery, oral nano-delivery system approaches, active-targeting ligands (folate, transferrin, aptamers, antibodies, and peptides) for nanocarriers, and future advances in colon targeted drug delivery. This review guided the research and development of protein ligand-based nanocarriers in the future. Liparulo et al. developed a solid lipid nanoparticle loaded with 1,2-benzoquinone RF-22c as the most powerful and selective competitive inhibitor of the enzyme 5-lipoxygenase. The nanoparticles showed improved sustained release properties and targeting abilities in monocrotaline rat model of pulmonary arterial hypertension. In particular, this nanoparticle could ensure high drug concentration around damaged arteries for significant therapeutic efficacy. This article guided the research and development of peptide-based nanocarriers in the future. Du et al. prepared galantamine pamoate-loaded poly (lactideco-glycolide) microspheres and analyzed their *in vitro* and *in vivo* release profiles. The results suggested the microspheres have a sustained release for about 24 days *in vitro* and stable plasma drug concentration for 17 days in rats. This work provided a potential way to prepare protein macromolecule-based microspheres for drug delivery. Chen et al. reviewed the current advances of phytochemical delivery through transferosome (phytosome) for transdermal drug delivery. This review suggested that, as a unique lipid-based nanocarrier, transferosome (phytosome) has the potential to tackle the challenges associated with phytochemicals for transdermal delivery. Especially, this review provided a promising way to prepare peptide-based nanocarriers for the delivery of transdermal drugs. Wang et al. analyzed the differences between two types of metalloenzymes with loaded iron ions. The results suggested the differences were caused by the depth of the active pockets and the relative position of the substrate. This work pointed out that the carrier structures should be carefully considered for the research and development of protein nanocarriers for metal ion loading. Han et al. explored the glycosylation of zein hydrolysate as a nanocarrier for lutein delivery. The results showed the nanocarrier had more trap efficiency and better physical and chemical properties than unglycosylated control. Therefore, glycosylated zein hydrolysate had a potential for the development of protein-based nanocarriers. This study provided a promising glycosylation way to improve the properties of protein-based nanocarriers. All these works could provide useful guides for protein-based nanocarriers to deliver nutraceutical, diagnostic, and therapeutic agents.

